# New advances in asymmetric organocatalysis

**DOI:** 10.3762/bjoc.18.28

**Published:** 2022-02-28

**Authors:** Radovan Šebesta

**Affiliations:** 1 Department of Organic Chemistry, Faculty of Natural Sciences, Comenius University in Bratislava, Mlynská dolina, Ilkovičova 6, 842 15 Bratislava, Slovakia

**Keywords:** asymmetric organocatalysis, covalent activation, noncovalent activation

Asymmetric catalysis is undoubtedly the most efficient way to prepare chiral compounds that our society requires as medicines, materials, or crop protecting agents. Traditionally, enzymes and metal complexes with chiral ligands served as the main type of enantioselective catalysts. Even though small chiral organic compounds have been recognized as chiral organocatalysts as early as 1912, the concept was at the periphery of the attention of synthetic organic chemists. An initial flash of interest appeared in the 1970s when proline was shown to catalyze the Robinson annulation [1–2], but this seminal work seemed to come too early to stimulate greater developments. Things started to change in the late 1990s when short-chain peptides [3], carbohydrate-based ketones [4–5], and thioureas [6] were shown to catalyze enantioselective transformations. The real breakthrough came in the year 2000 when two teams independently disclosed important discoveries with proline and imidazolidinones as ample chiral catalysts for aldol [7–8], Diels–Alder [9], dipolar cycloaddition [10], and Mannich reactions [11]. The organic chemistry community this time took a tremendous interest in this concept, which led to many valuable developments [12]. The recent culmination of the rapid advent of organocatalysis was the Nobel prize in 2021, which was awarded to Benjamin List and David MacMillan for their pioneering discoveries. Organocatalysis outgrew its initial inspiration by enzymatic catalysis, but the analogy with nature can be seen within the area. Organocatalytic reactions are highly suitable components of cascade transformations as exemplified by the seminal work of Enders [13]. Besides traditional enamine and iminium activation of carbonyl compounds other activation modes were uncovered which significantly broadened the repertoire of chemical transformations that are amenable to organocatalysis [14]. Within the realm of covalent activation, chiral carbenes and phosphines are diverse and structurally rich groups of catalysts. The synthetic scope was greatly expanded by noncovalent activation via a range of proton-mediated transformations using chiral Brønsted acids, Brønsted base, and hydrogen bond donors. Recently noncovalent activation continues to expand into other types of weak attractive interactions such as halogen and chalcogen bonds. Not surprisingly, all activation modes allow further expansion and diversification via a combination of activation modes in bifunctional or multifunctional catalysis. Important is also a “green” aspect of organocatalysis as well as its fruitful overlap with many sustainability ideas [15].

In 2012, there has been a thematic issue of the *Beilstein Journal of Organic Chemistry* devoted to asymmetric organocatalysis edited by one of the pioneers Benjamin List. After another decade, this thematic issue likes to survey new advances in this field. Three review articles and nine research papers showcase the diversity and breadth into which asymmetric organocatalysis has grown since then.

The suitability of asymmetric organocatalytic methods to assemble biologically relevant compounds is highlighted by a review article devoted to the syntheses of coumarin derivatives [16]. Conjugated additions of stabilized nucleophiles are the cornerstone of organocatalytic methodology. Recent advances in this area are covered by a review article devoted to aza-Michael reactions of amines and amides [17]. The evolution of the understanding of noncovalent activation modes led to the realization that anion-binding is a critical feature in many transformations. Halide anions are highly relevant and widely occurring within many reactions and a variety of organocatalysts can engage with them [18].

Nine excellent research articles within this special issue demonstrate the current state of the art in asymmetric organocatalysis. Chiral isothioureas became useful Lewis base catalysts for various transformations. Weinzierl and Waser employed an isothiourea catalyst for esterification-mediated kinetic resolution of paracyclophane derivatives with planar chirality [19]. Parida and Pan showed that a Michael reaction coupled with an acyl transfer reaction between α-nitroketones and 4-arylidenepyrrolidine-2,3-diones can produce a variety of enantioenriched 1,5-dihydro-2*H*-pyrrol-2-ones [20]. The development of any area is critically dependent on the understanding of underlying features and relationships. Slugovc and co-workers provide such mechanistic investigation of phosphine-catalyzed Michael additions [21]. Chiral cyclopropenimines exemplify Brønsted base organocatalysts that are useful for diverse reactions not easily accessible by other means. Here, Lambert and co-workers employed this type of catalyst in the formation of pyroglutamates via enantioselective Michael addition of amino ester imines [22]. Phase-transfer catalysis relies on the deprotonation of one of the substrates, but basic conditions may limit the applicability of this methodology. A unique base-free variant of chiral phase-transfer catalytic alkylation of 2-oxindoles was developed by Connon and co-workers [23]. Pentacarboxycyclopentadienes are a unique type of Brønsted acid catalyst that expanded the range of available acidities as well as molecular arrangements in acid-catalyzed reactions. Veselý and co-workers demonstrated that these catalysts are effective in the enantioselective aminalization of aldehydes with anthranilamides [24]. To explore new possibilities in combination of covalent and noncovalent activation, our group designed and synthesized *N*-sulfinylpyrrolidine-containing ureas and thioureas and applied them in Michael additions of aldehydes to heterocycle containing nitroalkenes [25]. Dubey and Chowdhury showed that 1,4-conjugate additions of nitromethane to β-silyl α,β-unsaturated carbonyl compounds catalyzed by bifunctional squaramide catalysts are effective under solvent-free conditions [26]. Zhai and Du demonstrated that asymmetric [3 + 2] annulation reactions of 2-isothiocyanato-1-indanones with barbiturate-based olefins are efficiently catalyzed by cinchona-based thiourea catalysts [27].

As guest editor of this thematic issue, I am grateful to all authors for their excellent contributions. I thank the referees for providing their expertise and time, and the whole team at the *Beilstein Journal of Organic Chemistry* for their great level of professionalism and support.

Radovan Šebesta

Bratislava, February 2022
